# The Use of Photodynamic Therapy in the Treatment of Endometrial Cancer—A Review of the Literature

**DOI:** 10.3390/ijms25168772

**Published:** 2024-08-12

**Authors:** Aleksandra Żołyniak-Brzuchacz, Edyta Barnaś, Dorota Bartusik-Aebisher, David Aebisher

**Affiliations:** 1 Doctoral School, Medical College of the University of Rzeszów, 35-310 Rzeszów, Poland; aleksandraz@dokt.ur.edu.pl; 2 Department of Midwifery, Medical College of the University of Rzeszów, 35-310 Rzeszów, Poland; ebarnas@interia.eu; 3 Department of Biochemistry and General Chemistry, Medical College of the University of Rzeszów, 35-310 Rzeszów, Poland; dbartusikaebisher@ur.edu.pl; 4 Department of Photomedicine and Physical Chemistry, Medical College of the University of Rzeszów, 35-310 Rzeszów, Poland

**Keywords:** endometrial cancer, photodynamic therapy, photosensitizers

## Abstract

Endometrial cancer is the most common malignant tumor of the female reproductive system. It develops in the mucous membrane lining the inside of the uterine body—the endometrium, through the abnormal and continuous growth of cancer cells originating from the uterine mucosa. In recent years, there has been a significant increase in the number of cases in European countries. Photodynamic therapy (PDT) is an innovative and dynamically developing medical procedure, useful in the treatment of cancer and non-cancer tissue conditions. The PDT reaction involves the activation of a photosensitizing substance with visible light, which in turn leads to the formation of free oxygen radicals, which contribute to the destruction of the cell. PDT is minimally invasive, has few side effects, and preserves organ anatomy and function. Both diagnostics and photodynamic therapy as modern methods of treatment are becoming more and more popular in many research units around the world. They are most often practiced and tested in in vitro experimental conditions. In clinical practice, the use of PDT is rare. Comprehensive cooperation between scientists contributes to taking steps towards obtaining new, synthetic photosensitizers, directing their physicochemical properties, and showing the impact on a given organism. This review examines the evidence for the potential and usefulness of PDT in the treatment of endometrial cancer. This review highlights that PDT is gaining popularity and is becoming a promising field of medical research.

## 1. Introduction

The photodynamic method is used in various fields of medicine, both in diagnostic processes (photodynamic diagnosis; PDD) and therapeutic processes (photodynamic therapy; PDT). The therapy uses three basic components: a photosensitizer, a light source, and oxygen dissolved in the tissue. The interaction of a specific wavelength with a photosensitizer determines its activation and causes a photodynamic reaction (the chemical compound and the light are individually non-toxic, but in combination they destroy tissues). The method is used in the diagnosis and treatment of pre-cancerous lesions and cancers of various types. PDT has a number of advantages. Photocytotoxicity effects are local and selective, as the photodynamic reaction occurs only as a result of irradiation. The operating procedure is simple and painless. It qualifies for outpatient treatment, which, in the case of oncological diseases, can be fully combined with other standard treatment methods, such as chemotherapy or radiotherapy. Depending on the area being treated, PDT may be minimally invasive [1]. Various publications describe the use of therapy in gynecological oncology. Some of the most common photosensitizers used in gynecology are protoporphyrin IX precursors. Minor side effects make PDT seem to be a promising and effective treatment method, as indicated by scientific reports. PDT is particularly interesting in the case of lesions located in accessible cavities, which include the endometrium.

## 2. Material and Methods

This literature review focuses on the use of photodynamic therapy in the treatment of endometrial cancer. A systematic approach was used to identify and select the studies, aiming to improve the quality of the manuscript. Articles were searched in the databases Medline (via PubMed), Library, Embase, and Web of Science. The search was performed using the terms: endometrial cancer, photodynamic therapy, uterine cancer, endometrium, photosensitizer, as well as the Boolean operators AND, OR, and NOT. The last search was conducted in March 2024. Only articles published in English and Polish were taken into account. A standard approach was used to collect data, including the first author of the article, year of publication, type of photosensitizer, and the main results.

## 3. History of Photodynamic Therapy

The origins of PDT took place over 100 years ago. Oscar Raab—a German medical student, performed an experiment involving the study of fluorescent dyes on infuroria (a collective term for freshwater microorganisms, including protists and microcrustaceans, including paramecia, euglena, cyclops, rotifers, and daphnia). As a result, he noticed that intense light exposed to the dye caused rapid degradation of microorganisms (he observed the death of Paramecium caudatum after exposure to light using acridine orange). Then, together with Professor Hermann von Tappeiner, an Austrian pharmacologist, he investigated the use of the PDT reaction in dermatology. In 1903, Doctor Jesionek also joined the experiment, and together they described the use of this new method also in dermatology (they used eosin and visible light topically in the treatment of skin cancer, condylomas, and lupus vulgaris) [2,3,4,5,6]. Eight years later, in 1911, Husmann performed tests on mice, giving them the substance and leaving them in sunlight. He further described hematoporphyrin. After two years, Friedrich MeyerBetz performed an experiment on himself, using 200 mg of hematoporphyrin intravenously and exposed himself to sunlight. The effect was increased swelling, pain, and itching at the site of exposure. In 1942, Auler and Banzer conducted experiments using porphyrins. A few years later, in 1948, Figge and Weiland were looking for solutions to use porphyrin derivatives in diagnosis and treatment using the photodynamic method [5]. The breakthrough came in 1976 when Thomas Dougherty reported the cure of a cancerous tumor in mice after using photodynamic therapy using a porphyrin derivative. Subsequently, studies were carried out on humans. The results turned out to be more and more promising. In 1978, the above-mentioned researcher proved the effectiveness of PDT in 25 patients (113 skin tumors were treated), in the vast majority of cases (98 cases) there was a complete cure, while in 13 people, a partial response was observed. The remaining two cases showed complete resistance [7,8,9].

## 4. Endometrium

The endometrium is the mucous membrane that lines the uterus. It is sensitive to the action of hormones (estrogens and progesterone), which lead to cyclical modifications that enable the implantation of the embryo or end with monthly menstruation. The basal layer of the endometrium contains epithelial and mesenchymal stem cells that have the ability to self-renew throughout reproductive life, ensuring cyclical reconstruction of the functional layer [10]. Estrogens are responsible for the proliferation of endometrial cells, regulating cell survival and viability, as well as mitogenic effects [11]. When the ovum is fertilized, the function of the endometrium changes. The fertilized egg attaches to the lining of the uterus. The placenta then develops. The placenta transfers nutrition and oxygen to the fetus from the mother. If the egg does not become fertilized, the lining of the uterus (endometrium) is shed during menstruation [12].

Moreover, the dysregulation and dedifferentiation of these phenomena exposes the endometrial glands to stimuli that play an essential role in proliferation and diseases—hyperplasia and cancer.

Endometrial abnormalities include benign and malignant lesions. Inappropriate uterine bleeding is a clinical manifestation of common benign pathologies, including adenomyosis and endometrial polyps.

## 5. Endometrial Cancer

Endometrial cancer is the most common malignant tumor of the female reproductive system in developed countries. It ranks 7th among all women’s cancers, and 4th place in Europe among women’s cancers (as of 2020), after breast, lung and colorectal cancer with an incidence rate of 12.9–20.2:100,000 and a low mortality rate of 2.0–2.7:100,000. This discrepancy is due to the fact that 80% of diagnoses are limited to the uterus at the time of diagnosis and manifest as postmenopausal bleeding, which allows for quick detection. It is diagnosed annually in approximately 6000 women in Poland, and approximately 1000 die from it. Racial, socioeconomic, and geographic differences are important factors that determine incidence and mortality. This cancer includes various histological subtypes and molecular phenotypes. Most cases occur between the ages of 65 and 75, with the peak incidence occurring in the 7th and 8th decade of life. In recent years, an increase in the number of cases has been observed in European countries [13]. The vast majority of endometrial cancers (80%) are diagnosed at an early stage, while some are detected in women under 40 years of age [14].

The endometrium is the mucosal layer of the uterus, and it is an extraordinary tissue, given its capability for monthly cyclical changes, capacity for growth, response to ovarian hormones, high number of receptors, and unique biochemical characteristics. The causes of mucosal disorders are generally bacteria, viruses, or fungi, such as yeast. A weakened immune system, stress, or dietary deficiencies can make someone more prone to a mucosal disorder [15,16].

## 6. Treatment

Cancer diseases often require invasive procedures—surgery, followed by radiotherapy or chemotherapy. The above-mentioned treatment methods often have a number of health consequences. Hysterectomy is a medically necessary procedure in the case of several conditions that threaten the life or health of patients, including endometrial cancer. However, this procedure is associated with health consequences, which include, among others, lowering of the pelvic organs and, consequently, problems with urination, such as increased frequency of urination, urinary incontinence, or recurrent urinary tract infections. Moreover, removal of the uterus causes disturbances in the statics of the pelvic organs. Problems related to sexuality may occur, such as decreased sexual sensations or lack of adequate vaginal lubrication during intercourse. Patients after hysterectomy may also experience symptoms of depression and low mental mood resulting from the feeling of loss of femininity. Importantly, hysterectomy involves removing the uterus, which excludes pregnancy.

Conservative methods are used in patients with comorbidities, significant obesity, women with specific features of cancer or genetic factors, as well as in patients of reproductive age. According to the literature, these methods are not fully effective. It has been proven that patients under 40 years of age have a higher rate of relapses [17,18], therefore, it is necessary to constantly search for new diagnostic and therapeutic methods of treating endometrial cancer. Endoscopic access to the uterus puts PDT in the spotlight in the treatment of endometrial diseases. Therefore, PDT may become an effective, conservative method of treating radical cases that do not qualify for surgical treatment and fertility-sparing cases in women.

## 7. Components Used in Photodynamic Therapy

The implementation of PDT and selective destruction of the cancerous area during photooxidation requires the use of three basic components. Photosensitizer—a chemical compound, a dye is one of the first ingredients which selectively accumulates in diseased tissue. The second key element is a light source that has the ability to excite a photosensitizer. The light emission band must match the absorption maximum of a given dye. The third necessary component for the reaction to occur is the appropriate amount of oxygen dissolved in the tissues. The mentioned therapy components are individually non-toxic, but their mutual combination initiates a strong photochemical reaction, leading to the destruction of cells showing signs of pathology [19].

## 8. The Importance of Light in Photodynamic Therapy

In PDT, light is one of the essential factors determining the course of therapy.

Properties of laser radiation are as follows:

- High monochromaticity;
- Small divergence, as a result of which high power density can be obtained;
- Coherence [20].

For PDT to be initiated, the photosensitizer consolidated in the tissue must absorb radiation of the appropriate wavelength. The absorption is weakened by endogenous dyes and water present in the tissue. It is important to use photosensitizers whose maximum absorption corresponds to the so-called medical tissue transmission window (area where the tissue absorption coefficient is lower for the indicated wavelength) [20]. The wavelength and tissue type determine the depth of radiation penetration. The penetration depth in melanotic tissues ranges from 0.2 mm for blue light to 0.6 mm for radiation from a wavelength of 900 nm, while in non-melanotic tissues the penetration depth is 0.5–1.5 mm [21]. In phototoxicity, people have pain and develop redness, inflammation, and sometimes brown or blue-gray discoloration in areas of skin that have been exposed to sunlight for a brief period. All phototoxic reactions appear only on areas of skin that have been exposed to the sun. Some plants (including limes, celery, and parsley) contain compounds called furocoumarins that make some people’s skin more sensitive to the effects of UV light. This reaction is called phytophotodermatiti. In photoallergies, an allergic reaction causes redness, scaling, itching, and sometimes blisters and spots that resemble hives. Substances that cause photoallergies are capable of doing so only after the person has been exposed to both the substance and sunlight (because sunlight is what makes the substance capable of triggering the photoallergy). Photoallergic reactions can also affect areas of skin that have not been exposed to the sun [22].

## 9. Mechanism of Action of Photodynamic Therapy

**Figure 1:**
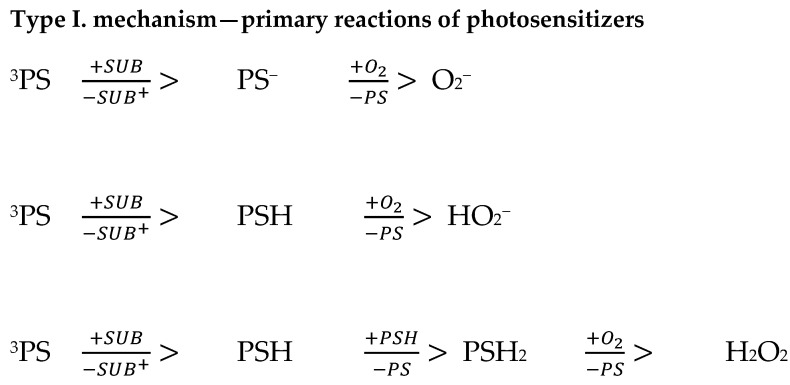
Formation of peroxide radicals and hydroxyl radical [25].

**Figure 2:**
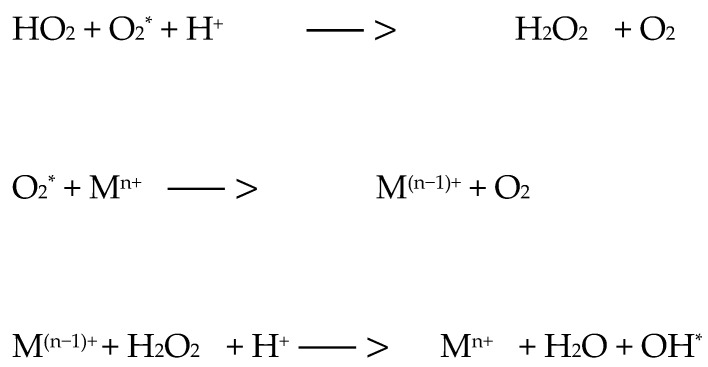
Possible reactions in the further course of therapy [25].

**Figure 3:**
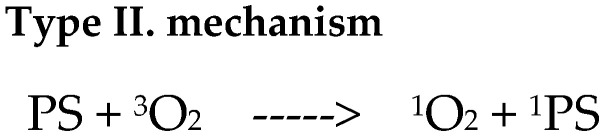
Formation of singlet oxygen [25].

There are two mechanisms of photooxidative reactions. A type I reaction involves the transfer of an electron or hydrogen atom, as well as the creation of a radical form of a photosensitizer or substrate (Figure 1). The listed ingredients are able to react with oxygen, resulting in the formation of peroxide forms, superoxide ions, and hydroxyl radicals; they can implement free radical chain reactions (Figure 2). However, in the type II reaction, energy is transferred between the photosensitizer molecule and the oxygen molecule [22,23,24,25]. As a result, the photosensitizer returns to the ground state and oxygen is excited to the singlet state (the ground state for the oxygen molecule—the triplet state). The generation of singlet oxygen has a fundamental role in the cytotoxicity process [23]. The amount of oxygen in the reaction environment determines the mechanism of the process. Reduced oxygen concentration is equivalent to a type I reaction (with the formation of free radical species). Superoxide ions and superoxide radicals are strong oxidants, which is why they contribute to the destruction of cancer. A type II mechanism occurs when the area where PDT occurs has molecular oxygen resources (Figure 3) [24,25]. The triplet state is the essential state for molecular oxygen. It is characterized by two unpaired electrons in p orbitals, and the total electron spin is S = 1. The singlet state, in turn, is characterized by two paired electrons and a resultant electron spin S = 0. Singlet oxygen is a metastable form characterized by high reactivity, which results in cell degradation through oxidative mechanisms similar to mechanism I [24,25].

For the markings used: 3PS—photosensitizer in the triplet state; 1PS i PS—photosensitizer in the ground state; SUB—reactive substrate and Mn+—metal ion

## 10. Photodynamic Therapy in the Treatment of Endometrial Cancer

**Figure 4:**
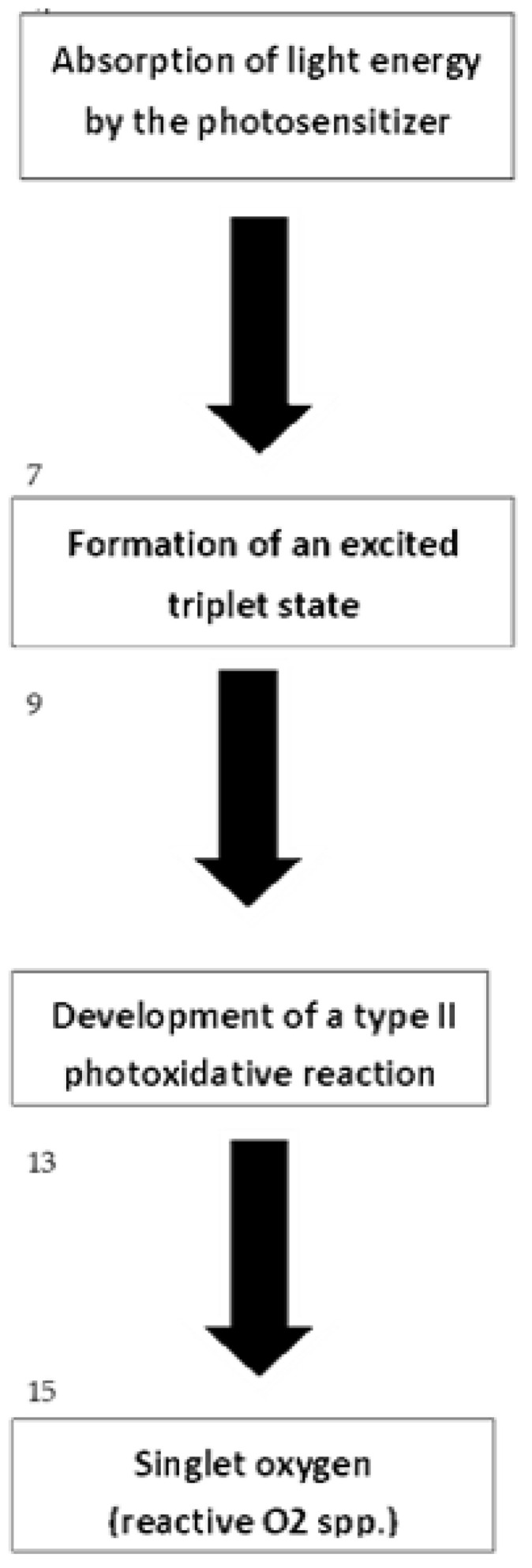
PDT course developed to show the main steps of reactions [26,27,28].

A fundamental experimental trend undertaken by scientists is the use of the PDT method in cancer treatment [26,27,28] (Figure 4).

Corti L. et al. presented a study in which they described the cases of 26 patients with gynecological cancers, including 5 with endometrial cancer—endometrial adenocarcinomas. The photosensitizer used was hematoporphyrin (HP) at a dose of 5 mg/kg body weight. The overall response was 2 of 7 for adenocarcinoma (28%). The study showed that PDT is effective and safe in the case of loco-regional recurrence of gynecological cancers (no deaths due to treatment and no serious complications were reported). It was emphasized that PDT still needs to be considered as a research tool (Table 1). To improve results, more stable and tumor-selective photosensitizers, a new laser system with higher tissue transmittance, and more precise tissue light dosimetry should be identified [29]. Koren et al. described the treatment of seven patients with early-stage endometrial cancer (FIGO 1a). Tumor irradiation was performed using an argon dye laser 24–72 h after intravenous administration of HPD (Phtotosan III, 2 mg/km body weight). Response to treatment was assessed 1 month after therapy. Most had a partial response or disease relapse within 12 months. The research results suggested that PDT may be a promising method for treating cancer. The authors emphasized that further research is necessary to develop possible therapy control systems in clinical trials [30]. Choi Y. et al. described a combination experiment combining photodynamic therapy with chemotherapy. The aim of the article was to investigate the molecular mechanism of the increased therapeutic efficacy of ccPDT as a result of the determination of intracellular ROS, as well as necrotic or apoptotic cell damage in HeLa cells loaded with fluorescent oxidizing agents and Photofrin and/or carboplatin using light rays. The combination of carboplatin with HPD resulted in greater cytotoxicity and the production of reactive oxygen species. While the concentrations of hydroxyl radicals and superoxide anions showed stable levels, a slight increase in hydrogen peroxide was seen after PDT application, and ROS concentrations also showed an increase in combination with carboplatin. PDT also increased the number of necrotic cells, while PDT combined with carboplatin increased the number of apoptotic and necrotic cells. The analysis showed that the combination with carboplatin showed better results when using low strengths ranging from 330 to 660 mJ. Photodynamic therapy has been proven to reduce unwanted side effects compared to standard treatments [31]. In the study by Ziółkowski et al., PDT was used in the treatment of G1 endometrial cancer tissues from patients with cancer. The women underwent total hysterectomy and bilateral salpingoophorectomy. To evaluate the possible role of laminin, a component of the basement membrane, as well as the susceptibility of the epidermal growth factor receptor to PDT, immunohistochemical studies were performed. The experiment confirmed that PDT causes necrosis of treated endometrial cancer without affecting laminin in the tumor tissue. Laminin may contribute to preventing the spread of cancer in cases where it is necessary to repeat PDT; after PDT, cells become less susceptible to a mitogen, such as epidermal growth factor [32]. Varriale L. et al. described the effect of hypericin using an endometrial cancer cell line and showed that photosensitization of HEC1-B cells with a low concentration of hypericin and light doses below 10 J/cm^2^ was associated with cell death, while the initial exposure of cells to harmless irradiation (2 J/cm^2^) and further challenge with a dose of light that normally induced apoptosis (5 J/cm^2^) changed protein expression in the regulation of apoptosis, the stress response, and the cell cycle. The research result—an increase in cell phototolerance [33]. Researchers Raab et al. conducted an experiment in which they studied human gynecological cancer cell lines HEC-1-A (endometrial cancer) using photodynamic therapy in vitro. A porphyrin compound called Photosan III was used to photosensitize cells after incubation times of 24 h. Scientists have proven that the endometrial cancer cell line is insensitive to hematoporphyrin derivative (HPD) at a concentration of up to 10 µg/mL after 24 and 48 h of incubation, when there is no access to light. The use of photodynamic therapy led to complete eradication of the cells by treatment with a stronger dose of 10 µg/ mLHPD with a 24-h incubation period or 5 g/mL with a 48-h incubation period, which were consequently predisposed to treatment with limited secondary effects [34]. In the article by Schneider-Yin X. et al., a study was conducted on the human endometrial adenocarcinoma cell line HEC-1A using 5-aminolevulinic acid (5-ALA) and hypericin. As a result of the experiment, it was observed that 5-ALA reduced cell survival, but no improvement was noted with the combination of both photosensitizers after irradiation with a wavelength of 635 nm. Researchers showed that the combination of hypericin and 5-ALA-stimulated PpIX resulted in higher phototoxicity against human endometrial cancer cells using an incoherent white light source [35]. The purpose of Kim Su-Mi’s research was to investigate the effects of radachlorin-based photodynamic therapy on invasion, vascular formation, and apoptosis by targeting epidermal growth factor receptor (EGFR)/vascular endothelial growth factor receptor 2 (VEGFR2) signaling pathways in the endometrial HEC-1-A adenocarcinoma cell line. Researchers demonstrated that PDT exerted antitumor effects on HEC-1-A by activating the intrinsic apoptosis pathway via caspase-9 and poly(ADP-ribose) polymerase (PARP). PDT also inhibited tubular capillary formation and HEC-1-A invasion during VEGF pretreatment. The main advantage of PDT with radachlorin is its selectivity towards tumor tissue while preserving adjacent normal endometrial tissue. Therefore, radachlorin-mediated PDT may provide high antitumor efficacy in endometrial adenocarcinoma and be a particularly useful fertility preservation method [36]. The study by Choi et al. assessed the effectiveness of PDT as a conservative method of fertility-sparing treatment in young women with early-stage endometrial cancer. In total, 16 women were included in the analysis. The medical records of patients with endometrial cancer who were treated with PDT were reviewed. People under 35 years of age with early-stage disease were included. A photosensitizer of the hematoporphyrin derivative type Photogen was used at a dose of 2 mg/kg 48 h before the use of laser light. PDT was used in 11 patients as the primary treatment, and in 5 patients as a secondary treatment in the event of relapse after primary hormonal therapy. Complete remission was observed in 12 (75%), of which 4 (33%) relapsed, and the results were comparable to hormonal therapy and were achieved faster. However, after the second cycle of PDT, complete remission was observed in one woman with a relapse of the disease and in one woman who did not respond to treatment. In total, 57% of women became pregnant, mainly through assisted reproductive technologies [37]. Choi et al. described the case of a 31-year-old woman with low-grade endometrial sarcoma. The woman underwent laparoscopic lymphadenectomy, polypectomy, cervical curettage, and HPD-PDT (endometrium and cervix). Due to the risk of disease recurrence after conservative surgery, adjuvant treatment with a non-steroidal aromatase inhibitor (letrozole 2.5 mg, orally daily) was also included for a period of 6 months. Subsequent clinical examinations and radiographic examinations showed no recurrence of the disease in the patient for 99 months. After 32 months, as a result of in vitro fertilization, the woman became pregnant with twins. Preterm delivery occurred at 32 + 2 weeks of gestation by cesarean section. Placental pathology showed no signs of cancer [38].

PDT treatment covers only the diseased spot—a point, leaving the health of the tissue intact (only the pathological tissue is destroyed, thanks to the accumulation of photosensitizer in it in a higher concentration than in the surrounding tissues and precise exposure of the pathological tissue using a lamp) [26]. 

PDT is a technique in which visible light is used in combination with photosensitizing agents to obtain a carcinogenic effect. The use of PDT in gynecology is limited. Several investigators have reported mixed results in the treatment of lower genital tract intraepithelial tumors and recurrent malignancies using various modalities including PDT. A limitation of PDT in endometrial cancer is the lack of compounds that are both good PS and good tumor localizers. PDT with complex anatomy often causes significant toxicity to surrounding healthy tissues. Techniques such as photobleaching and the use of photosensitizers with weak absorption bands at lower wavelengths may reduce cutaneous toxicity in the future. Light for PDT is typically provided by argon-pumped dye lasers or metal vapor lasers. Diode lasers will be used in the future. The use of optical fibers and scattering lenses enables the endoscopic and interstitial use of PDT. The mechanism of action of PDT is the creation of singlet oxygen, which oxidizes biological molecules and causes irreversible subcellular damage. The main effect of PDT in vivo is due to the destruction of the tumor vasculature, causing anoxia and necrosis. However, eligibility criteria are sometimes more restrictive than necessary, and expanding eligibility criteria to be more inclusive is one study design issue that can improve the diversity of clinical trial populations; eligibility criteria vary for each study. These include whether you are a healthy or patient volunteer. They also include factors such as your age and gender, the type and stage of your disease, whether you have had certain treatments, and whether you have other health problems.

## 11. Conclusions

This review highlights a promising area of research in the treatment of endometrial cancer. Conservative treatment may involve combined multimodal therapies with a hysteroscopic approach, eradication of potential tumor remnants using PDT, and inhibition of recurrence as a result of adjuvant therapy. PDT is a dynamic area of research with great potential for development and may become an important treatment option for a wide range of oncological diseases. The limitations of the use of photodynamic therapy result from the insufficient number of randomized clinical trials and detailed treatment guidelines for given diseases, as well as the high costs of treatment. In Poland, insufficient availability of photosensitizers and their high price are two of the factors for not using the therapy. Promoting innovative methods for treating oncological diseases is crucial. PDT offers potential effectiveness, selectivity, and a less invasive approach to the treatment of many oncological diseases. It is important to educate the public about PDT in order to make treatments more effective. Moreover, the growing awareness of the population will contribute to the further development of this treatment method, which may result in a more widespread use of this method of therapy in the future in the treatment of oncological diseases.

## Figures and Tables

**Table 1:** Endometrial cancer PDT advantages, PDT disadvantage, contraindications to PDT treatment, and evidence for endometrial cancer prevention strategies.

PDT for Endometrial Cancer	
The most common cause of the development of the disease is	Excessive estrogen stimulation (both endogenous and exogenous), with simultaneous deficiency of progestogens
	Excessive body weight
	A family history of endometrial cancer, colorectal cancer or breast cancer
	Hormonal disorders caused by ovarian tumors
	Postmenopausal age
	Early menarche
	Late age of last menstrual period no offspring
	Occurrence of anovulatory cycles
	Polycystic ovary syndrome
	Diabetes
	Presence of congenital predisposition syndromes (Lynch syndrome, Cowden syndrome)
PDT Advantages	Fewer adverse effects
	Low invasiveness
	Short treatment time
	Usable in outpatient settings
	Double selectivity
	Can be applied in the same time
	Little or no scar after healing
	Lower cost than other treatment
PDT Disadvantages	Photosensitivity after treatment
	Treatment efficacy depends on accurate light delivery to the tumor
	Tissue oxygenation is crucial to the photodynamic effect
	Impossible to treat metastatic cancers with current technology
Contraindications to PDT treatment	A non-responsive tumor
	Porphyria
	Systemic lupus erythematosus
	Other photosensitivity dermatoses
	Allergy to the photosensitiser (very rare)
Evidence for endometrial cancer prevention strategies	Obesity is an established risk factor for endometrial cancer
	Weight cycling and weight gain in middle age are risk factors for endometrial cancer.
	Bariatric surgery reduces the risk of endometrial cancer by up to 81% in obese women who attain and maintain a normal weight.
	Combined oral contraceptives provide durable protection against endometrial cancer for 30 years or more.
	Ever use of the levonorgestrel intrauterine system (LNG-IUS) and inert intrauterine devices reduce endometrial cancer risk.
	The first oestrogen-based non-progestin HRT for non-hysterectomised women that contains estradiol and bazedoxifene has an effective protective effect on endometrium.
	Weight cycling and weight gain in middle age are risk factors for endometrial cancer.

## Data Availability

All data is included in the manuscript.
